# Exclusivity offers a sound yet practical species criterion for bacteria despite abundant gene flow

**DOI:** 10.1186/s12864-018-5099-6

**Published:** 2018-10-03

**Authors:** Erik S Wright, David A. Baum

**Affiliations:** 1 Department of Biomedical Informatics, University of Pittsburgh, Pittsburgh, USA; 2 Pittsburgh Center for Evolutionary Biology and Medicine, Pittsburgh, USA; 3 Department of Botany, University of Wisconsin-Madison, Madison, USA

**Keywords:** Horizontal gene transfer, Phylogeny, Species, Taxonomy, Classification

## Abstract

**Background:**

The question of whether bacterial species objectively exist has long divided microbiologists. A major source of contention stems from the fact that bacteria regularly engage in horizontal gene transfer (HGT), making it difficult to ascertain relatedness and draw boundaries between taxa. A natural way to define taxa is based on exclusivity of relatedness, which applies when members of a taxon are more closely related to each other than they are to any outsider. It is largely unknown whether exclusive bacterial taxa exist when averaging over the genome or are rare due to rampant hybridization.

**Results:**

Here, we analyze a collection of 701 genomes representing a wide variety of environmental isolates from the family Streptomycetaceae, whose members are competent at HGT. We find that the presence/absence of auxiliary genes in the pan-genome displays a hierarchical (tree-like) structure that correlates significantly with the genealogy of the core-genome. Moreover, we identified the existence of many exclusive taxa, although individual genes often contradict these taxa. These conclusions were supported by repeating the analysis on 1,586 genomes belonging to the genus *Bacillus*. However, despite confirming the existence of exclusive groups (taxa), we were unable to identify an objective threshold at which to assign the rank of species.

**Conclusions:**

The existence of bacterial taxa is justified by considering average relatedness across the entire genome, as captured by exclusivity, but is rejected if one requires unanimous agreement of all parts of the genome. We propose using exclusivity to delimit taxa and conventional genome similarity thresholds to assign bacterial taxa to the species rank. This approach recognizes species that are phylogenetically meaningful, while also establishing some degree of comparability across species-ranked taxa in different bacterial clades.

**Electronic supplementary material:**

The online version of this article (10.1186/s12864-018-5099-6) contains supplementary material, which is available to authorized users.

## Background

It has long been debated whether bacterial taxa, in particular species, are real entities [1]. Many have argued for the existence of ecologically [2, 3], phenotypically [4], or genetically [5–7] distinct groups of bacteria that reflect real discontinuities in nature [8–10]. Others maintain that the combination of high dispersal rates and rampant horizontal gene transfer (HGT) has resulted in bacteria spanning a continuous spectrum of types without natural divisions and that, therefore, the notion of species does not apply [11, 12]. At stake in this debate is our fundamental understanding of how bacterial life is organized, which has important medical and research implications [13]. Moreover, since HGT (including sexual hybridization) occurs to some degree in all kinds of living organisms, clarifying the nature of bacterial species has significant implications for the entire taxonomic enterprise.

The rapid rise in the number of available bacterial genomes has offered many insights into bacterial evolution and has the potential to shed new light on the nature of bacterial species. Studies of conserved genes in many bacterial groups have discovered abundant admixture due to homologous recombination, revealing a complex network of gene flow that may be incompatible with a hierarchical Linnaean taxonomy [14, 15]. In reaction to such findings, it has been argued that there exists a subset of core-genes that are more resistant to HGT and exhibit greater vertical inheritance [16]. Species can then be delineated as clusters on a core-genome tree whose members regularly exchange DNA with one another but not with organisms outside the cluster, analogous to the traditional biological species concept [7, 17–19]. However, this approach has been criticized for trying to force a bifurcating tree onto what is really a reticulate network [12]. Additionally, the idea that HGT drops off abruptly at the rank of species is contradicted by numerous well documented cases of gene transfer among more distant relatives [20, 21].

While much focus has been placed on the effects of homologous recombination, bacteria also undergo considerable non-homologous recombination, which allows them to quickly acquire auxiliary genes that may be the primary determinants of their ecological specialization, that is, ecotype [22]. In contrast to core-genome approaches, pan-genome approaches typically focus on the presence or absence of auxiliary genes [23, 24]. The fact that pan-genomes are highly labile has been used to cast further doubt on the applicability of the species category in bacteria. For example, strains assigned to the traditional species *E. coli* may have only 40% of their genes in common, which has been taken as evidence against the existence of bacterial species [12]. However, this argument relies on the notion that current species assignments are correct, and would easily be refuted if *E. coli* were actually an assemblage of multiple real species. Thus, it is desirable to clarify whether species exist in a manner that is independent of the groups that have been treated as species historically.

Whether considering micro or macro organisms, the definition of species is notoriously controversial, and a variety of alternative species concepts have been proposed [25, 26]. Whereas many species concepts focus on identifying lineages that are perceived to directly participate in evolution, for this study we took a strictly genealogical approach, focusing only on the challenge of grouping the diversity of currently living organisms into taxa based on their degree of evolutionary relatedness. Specifically, we explored the position that species, like other ranks in the taxonomic hierarchy, are groups of contemporaneous organisms that have the property of *exclusivity:* all members of the group are more closely related to each other than to any organism outside the group [27–30]. Exclusivity is preferable to monophyly as an evolutionary species concept because monophyly is undefinable at the genome level when different parts of the genome have different gene trees [27], whereas exclusivity can be applied by averaging relatedness across the genome. It is largely unknown whether exclusive taxa of bacteria exist, since historical HGT can create intermediates between different types that degrade exclusivity [31]. Only after determining whether exclusive taxa exist is it possible to ask whether there is an objective basis for assigning some exclusive taxa to the rank of species.

To address whether exclusive bacterial taxa exist we need to estimate the average degree of relatedness for a set of closely related bacterial genomes. Sequence divergence can be used as a first approximation of time since common ancestry (i.e., relatedness) for each orthologous gene shared by a pair of taxa. By averaging these distances across genes, we can obtain a genome-wide measure or pairwise genome relatedness. To evaluate such an approach, we need genomes from a broad sampling of strains belonging to a single supraspecific clade. In this context, an ideal bacterial genus is *Streptomyces*, which has genomes available for more named species than any other bacterial genus [32, 33]. The acquisition of diverse *Streptomyces* genomes, largely motivated by their immense capacity for producing secondary metabolites (e.g., antibiotics), has been relatively unbiased toward any named species [34] making *Streptomyces* an excellent system for exploring the bacterial species problem [35].

Streptomycetes are unusual among bacteria in having large (6 to 13 Mbp) linear genomes with conserved (central) and variable (outer) regions [36]. Strains of *Streptomyces* are competent at HGT [19, 35, 37]. Here we analyze the genomes of 701 strains belonging to members of the family Streptomycetaceae, including 676 *Streptomyces*, 15 *Kitasatospora*, and 10 *Streptacidiphilus*. We included multiple genera within this family because Streptomycetaceae strains are sometimes misclassified into the wrong genus [33]. For comparison, we also analyzed 1,586 genomes belonging to members of the genus *Bacillus*. While *Bacillus* encompasses fewer named species and is more biased towards clinically-important taxa, this data set provides an opportunity to evaluate whether patterns observed in Streptomycetaceae are shaped by the specialized biology of that group or are likely to apply to the majority of bacteria.

For both bacterial groups, we find that a very similar phylogenetic signal is shared by the core- and pan-genomes, suggesting that HGT has not overwhelmed vertical inheritance in these two bacterial groups. Based on the core-genome, we also identified numerous exclusive clades that persist despite widespread HGT on an individual gene basis. However, there does not appear to be a clear cutoff in exclusivity at which to assign taxa to the rank of species. In response, we proposed a methodology for designating species that uses conventional similarity-based thresholds to determine which exclusive groups are assigned to the species rank. This approach allows for high-throughput delimitation of exclusive species despite HGT.

## Methods

### Genome dataset

A total of 824 complete and draft genomes belonging to strains assigned to the family Streptomycetaceae were downloaded from GenBank on December 23, 2016. Similarly, 1,919 genomes labeled as *Bacillus* were downloaded from GenBank on September 14, 2017. Prediction of open reading frames was performed with Prodigal v2.6.2 [38].

### Clustering of protein sequences

The process of obtaining clusters of orthologous genes (COGs) from a genome’s protein coding sequences is summarized in Additional file 1: Figure S1. Shared homologs between each pair of genomes were identified using protein BLAST v2.2.31+ with an E-value reporting threshold of ≤1e-3 [39]. Since E-value is a poor predictor of functional similarity, the Homology-derived Secondary Structure of Proteins (HSSP) distance was used to filter very low quality matches. The HSSP distance is a non-linear function of the percent identity and local alignment (match) length. An HSSP distance of ≥20 and a match length of at least 30% was required for inclusion in the list of candidate homologs [40, 41]. To further improve accuracy, only reciprocal best BLAST hits were allowed to connect pairs of proteins between strains [42]. Finally, a minimum of 60% global amino acid identity was required to consider two connected proteins homologous [43].

The set of all homologous proteins was then clustered using MCL (Markov Clustering Algorithm) [44] with the inflation parameter (-I) set to 1.8 [45], which was shown in a previous study to result in similar conclusions to other values of the inflation parameter [43]. These MCL clusters were used to generate a binary matrix specifying the presence or absence of 286,312 COGs across all 824 Streptomycetaceae genomes. On average, 98.5% of genes belonging to a COG were present in a single-copy per genome. The subset of 74 strains with completed genomes each contained 5,163 to 9,165 COGs, of which 927 were shared by all 74 strains. Rather than ignoring the draft genomes, we reduced the set of 824 genomes to the 701 that contained at least 887 (95.7%) of the 927 COGs shared by all the complete genomes. The final matrix consisted of 235,145 COGs from 701 strains with complete or nearly complete genomes. A subset of 157 COGs were identified that are shared by all 701 strains and were defined here as the core-genome, while all other COGs were considered to comprise the pan-genome. By a similar procedure, the matrix of 1,919 *Bacillus* genomes, which generated 140,300 COGs, was reduced to the 1,586 genomes that shared 155 core genes.

### Construction of phylogeny based on core-genes

To construct a concatenated alignment of the 157 orthologous genes shared by all 701 Streptomycetaceae genomes, and 155 orthologous genes shared by all 1,586 *Bacillus* genomes, it was necessary in a small number of cases to choose from multiple gene copies associated with a particular COG. In such cases, the gene copy chosen was that with (i) the fewest degeneracies (i.e., Ns), (ii) lowest average pairwise distance to the other genes in the COG, and (iii) length closest to the median length of all genes associated with the COG. Notably, the vast majority of COGs contained relatively few genomes (< 1%) that required choosing a representative gene out of multiple copies.

Nucleotide sequences were aligned based on their amino acid translations using the *AlignTranslation* function in the DECIPHER package (v2.0) in R [46, 47]. Sites (columns) in the alignment with more than 90% gaps (insertions or deletions) were discarded. The resulting Streptomycetaceae concatenated alignment had a combined length of 141,717 sites, whereas the *Bacillus* genomes yielded a concatenated alignment with 150,363 sites. Maximum likelihood trees for individual genes and the concatenated alignments were estimated using RAxML (v8.1.20) [48] with the GTR + Γ model of molecular evolution. Patristic distances between every pair of strains on each gene tree were calculated using the *cophenetic* function in the R package APE (v5.1) [49]. These gene-specific pairwise distances were then averaged across genes to generate an overall distance measure that was used as a proxy for relatedness of each pair of taxa. The resulting distance matrix was clustered to yield a UPGMA tree using the DECIPHER package [50]. UPGMA was chosen because it is guaranteed to find all exclusive groups in a distance matrix [30].

To estimate exclusivity for clades that appeared on the core-genome UPGMA tree, we calculated the minimum distance between any member of the group and any genome outside the group and then subtracted the maximum distance between any two genomes included in the group. Exclusive groups, by definition, are those with a positive value of this exclusivity factor.

### Comparison of core-genome and pan-genome trees

The pan-genome was defined to include all COGs that are present in at least two genomes. For comparison to the core-genome patristic distances, we generated a pan-genome distance matrix based on gene presence-absence data using the “binary” (Jaccard distance) method of the *dist* function in R. This distance metric is bounded between 0 and 1 and avoids counting shared gene absences as evidence of relatedness. A UPGMA tree was generated from this matrix using the DECIPHER package in R [50]. We evaluated exclusivity from the core-genome average patristic distances for all clades present on the pan-genome UPGMA tree. The Robinson-Foulds (RF) distance between trees estimated from the core- and pan-genomes was determined using the *dist.topo* function from the APE package in R. The expected distribution of RF distances for random trees was computed using the equations provided in [51].

### Comparison to existing species delimitation criteria

Two genes commonly used in strain typing, the small subunit ribosomal RNA (16S rRNA) gene and DNA-directed RNA polymerase subunit β (*rpoB*) gene, were extracted from the genomes by searching for conserved flanking regions. Sequences with more than 10 degeneracies (i.e., Ns) were removed, as these may significantly distort the results by making organisms appear more closely related than in actuality. Finally, the two sets of genes were separately aligned using the DECIPHER package [46]. Average nucleotide identity (ANI) and alignment fraction (AF) were obtained for all pairs of genomes using the ANIcalculator (v1) [52].

## Results

### Streptomycetaceae genomes have highly variable gene content

We began by assembling the set of all Streptomycetaceae genomes publicly available from NCBI. We grouped proteins into COGs based on reciprocal best BLAST hits having pairwise amino acid identity of at least 60% (Additional file 1: Figure S1). This process resulted in identification of only 157 core genes shared by 701 complete or nearly-complete Streptomycetaceae genomes, as contrasted with 235,145 COGs comprising the pan-genome (see Methods). To explore pan-genome evolution, for each pair of genomes we calculated their protein-coding gene content similarity (the fraction of shared COGs; also known as proteome similarity) using the Jaccard index, a measure of binary similarity. Impressively, proteome similarity between pairs of Streptomycetaceae strains could be as low as 12.3%, with a median of 27.7% shared COGs. Even strains assigned the same species name had as little as 24.2% average gene content similarity and a median similarity of just 81.2%. The existence of divergent proteomes sharing the same species name justifies ongoing efforts to reclassify *Streptomyces* strains [33, 53, 54].

### Correlations with conventional species similarity criteria

**Fig. 1:**
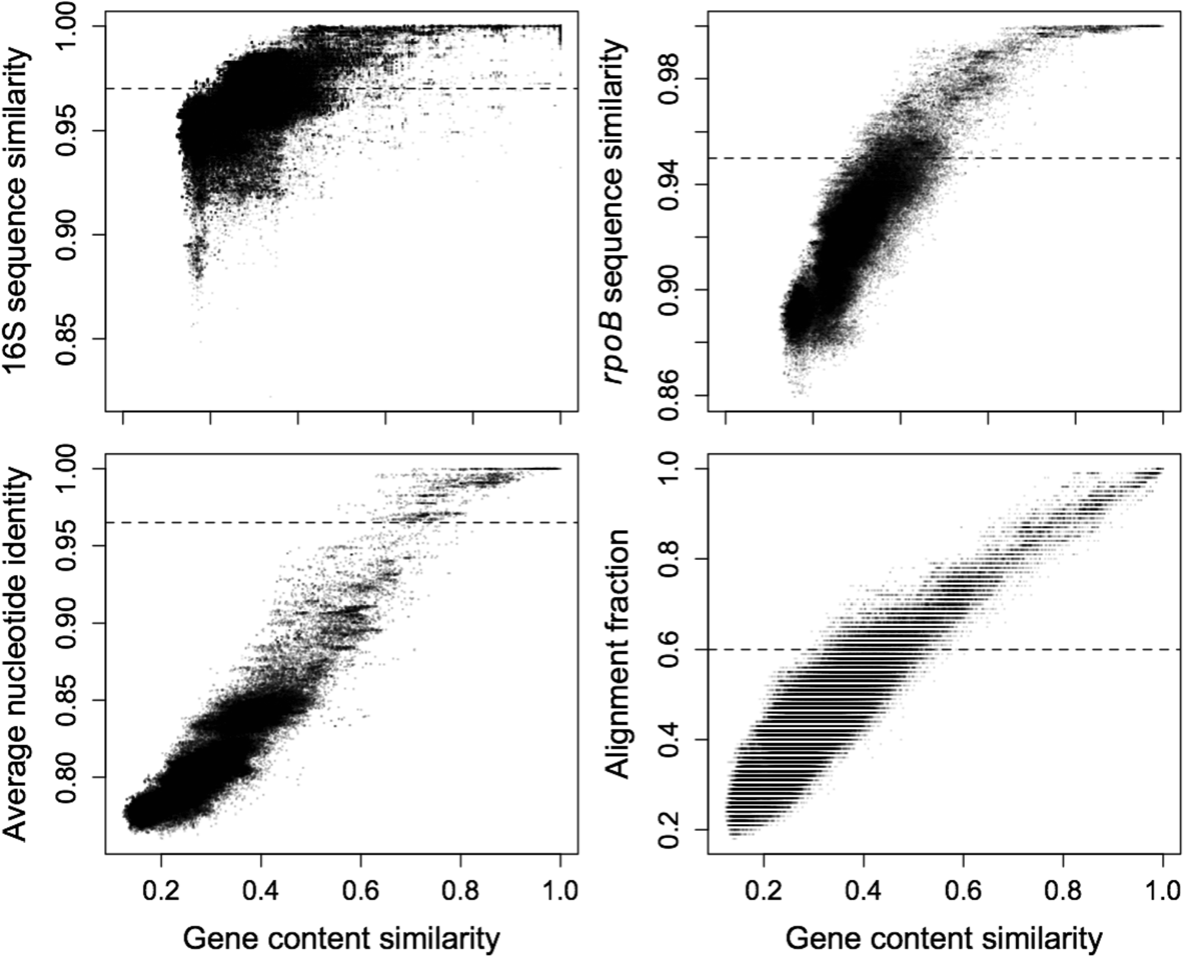
Common measures of the species rank exhibit widely different correlations with gene content similarity in Streptomycetaceae. The 16S rRNA sequence showed little correlation with gene content similarity (R^2^ = 0.38), whereas the *rpoB* gene sequence displayed good correlation (R^2^ = 0.75). Two genome-wide measures of similarity, average nucleotide identity (ANI) and alignment fraction (AF), were strongly correlated with gene content similarity. Horizontal dashed lines denote commonly used species-level cutoffs for each measure (e.g., > 97% 16S sequence identity)

A common approach to delimit species of bacteria is to use a cutoff of > 97% sequence similarity in 16S rRNA gene sequence. We found 16S rRNA similarity to be a poor predictor of proteome similarity (R^2^ = 0.38). For example, strains with identical 16S sequences varied between 39 and 100% in proteome similarity (Fig. 1). The low correlation between 16S and proteome similarity is likely a result of the limited size of the 16S rRNA gene and its slow rate of sequence evolution relative to the rate at which auxiliary genes are gained or lost. The poor predictive value of 16S sequences corroborates previous reports that, regardless of threshold, 16S rRNA similarity cannot be employed to reliably identify species [13, 16, 55–58], although it is still widely used for this purpose (e.g., [59]).

The *rpoB* gene has been proposed as a better species-level phylogenetic marker for *Streptomyces* because it exhibits a strong correlation with the results of multi-locus sequence typing [60]. Similarity in *rpoB* sequence showed good correlation (R^2^ = 0.75) with proteome similarity, but provided little resolution above 75% proteome similarity, where *rpoB* sequences are nearly identical (Fig. 1). This strong correlation was impressive, given that *rpoB* is a single gene and is believed to undergo homologous recombination between strains [19]. As might be expected, two genome-wide measures of similarity, ANI and AF [52], also displayed strong correlations with proteome similarity (R^2^ = 0.87 and 0.80, respectively).

### Pan-genome content shows signals of both horizontal and vertical inheritance

**Fig. 2:**
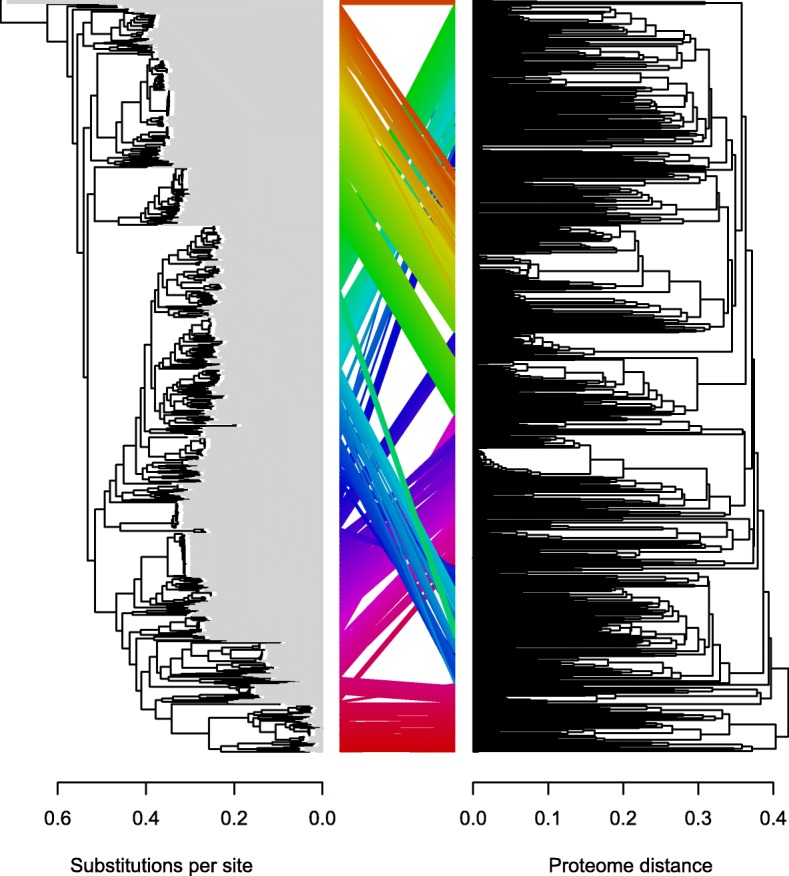
Strong correspondence between trees based on the core-genome and pan-genome in Streptomycetaceae. A maximum-likelihood tree from the core-genome (left) and a UPGMA tree based on gene content similarity (right) are similar in structure. The same 701 tips on both trees are connected by colored lines showing the degree of entanglement. Note that tips on the maximum likelihood core-genome tree (left) are extended by gray lines to the same horizontal position because the tree is not ultrametric like the pan-genome UPGMA tree (right)

We next sought to determine whether the core- and pan-genomes shared the same predominant genealogical history, as has been demonstrated in studies of other bacterial groups [61, 62]. To accomplish this, we built trees from these non-overlapping data partitions using distinct tree-building methods. For the core-genome, we computed a maximum likelihood tree based on a concatenated alignment of the 157 core genes. For the pan-genome, we constructed a UPGMA tree from the matrix of gene content similarities. Figure 2 shows that these two trees differed in the resolution of basal nodes but were nonetheless highly congruent, especially in their branching order within smaller clades, as seen in studies of some other bacterial groups [63, 64]. The RF distance between the two trees is 598, much lower than the distance expected for random trees (~ 1396); the probability of two random trees being this similar by chance is 10^− 1230^. The remarkable congruence between the two trees strongly suggests that both have been shaped by the same, predominantly tree-like, genealogical history.

Genes that were not included in the core-genome must have experienced at least one gain or loss event within the Streptomycetaceae. Although Fitch parsimony is an imperfect method, given its prior assumption that gains and losses are equally likely [65], it does provide a minimum estimate of the total number of gene gains/losses. Mapping the pan-genome onto the core-genome tree using Fitch parsimony [66] requires 1,113,832 changes, of which 850,208 are gains and 263,624 are loses (under ACCTRAN optimization). Since there are 1,399 branches on the tree, this implies an average of about 608 gene gains and about 188 gene losses per branch, consistent with prior findings that there is a considerable amount of horizontal gene transfer within *Streptomyces* [19, 35]. Nonetheless, the fact that the core- and pan-genome trees agree significantly despite such a high frequency of HGT (and other causes of discordance), suggests that HGT usually involves a diversity of donor and recipient lineages, which allows an overall signal of vertical inheritance to prevail.

### Exclusive taxa exist despite HGT

Species, like other ranks in the taxonomic hierarchy, are expected to meet the requirement of exclusivity, in which group members are more closely related to each other than they are to any non-group members [27, 67]. Given the shared genealogical history of the core- and pan-genome, we hypothesized that exclusive groups of bacteria may exist. Previously, the assessment of exclusivity has been based on the concordance factor: the proportion of the genome for which a given clade applies [27, 29]. The concordance factor treats all gene trees that lack a clade equally, regardless of how distantly related a group of tips are for each gene. Additionally, there is no specific concordance factor at which a group becomes exclusive: a group’s exclusivity depends on its concordance factor being greater than that of any conflicting group, which cannot be determined without checking the concordance factor of all clades with overlapping content. Here we developed a new approach based on the patristic (i.e., cophenetic) distances between each pair of strains. Since patristic distances differed across core-genes (mean R^2^ = 0.44), we averaged the patristic distances across all genes in the core-genome. This allowed us to quantify the degree of exclusivity as the minimum out-group patristic distance minus the maximum in-group patristic distance. Clades with a positive exclusivity score are exclusive, with higher scores indicating a greater degree of exclusivity.

**Fig. 3:**
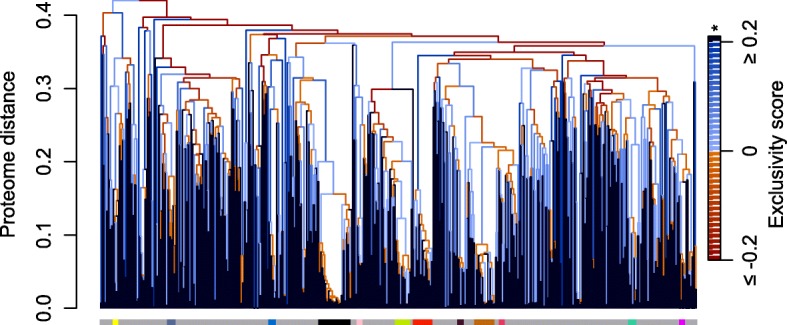
UPGMA tree of gene content similarity showing the distribution of exclusivity among 701 Streptomycetaceae genomes. Internal edges of the tree are colored based on their degree of exclusivity (see Methods), from not exclusive (orange) to exclusive (blue). Leaves of the tree are shown in dark blue because they are trivially exclusive (*). The horizontal bar below leaf tips is colored according to the 12 largest species (otherwise gray), here defined as single-linkage groups having AF > 0.6 and ANI > 96.5%. Exclusive groups are more frequent at lower taxonomic levels, which suggests either a lack of consistent signal to resolve deep relationships or true non-exclusivity due to wide hybridization events

Next, we compared the degree of exclusivity across three alternative approaches for defining groups, all while using the same matrix of average patristic distances to calculate exclusivity. First, we defined clades based on the pan-genome (gene content) tree. This was the most conservative approach because the data partition and analysis method used to delineate clades is distinct from that used to quantify exclusivity. For these data, exclusivity scores tended to be greater for clades with higher concordance factors, although larger clades were occasionally exclusive despite low concordance (Additional file 1: Figure S2). As might be expected, the exclusivity scores of internal edges were correlated with edge length on the pan-genome tree, with longer edges generally denoting clades having greater exclusivity (Additional file 1: Figure S3). In total, 358/699 (51.2%) of the internal edges on the pan-genome tree subtend exclusive taxa (as determined from the core-genome), and all but 41 of the 701 strains belong to at least one non-trivial (i.e., including > 1 strain) exclusive taxon (Fig. 3). The 41 strains that are not part of any multiply-sampled exclusive group all lack a close relative in the dataset, and therefore could reasonably be considered exclusive taxa by themselves (i.e., singletons). Second, we calculated exclusivity on the tree derived from a concatenated alignment of the 157 core genes, finding that 64.2% of clades were exclusive. Third, we used UPGMA to infer a tree based on the average patristic distances themselves, finding that 71.5% of clades on this tree were exclusive. Taken together, these three approaches demonstrate that exclusive clades are abundant among Streptomycetaceae genomes.

**Fig. 4:**
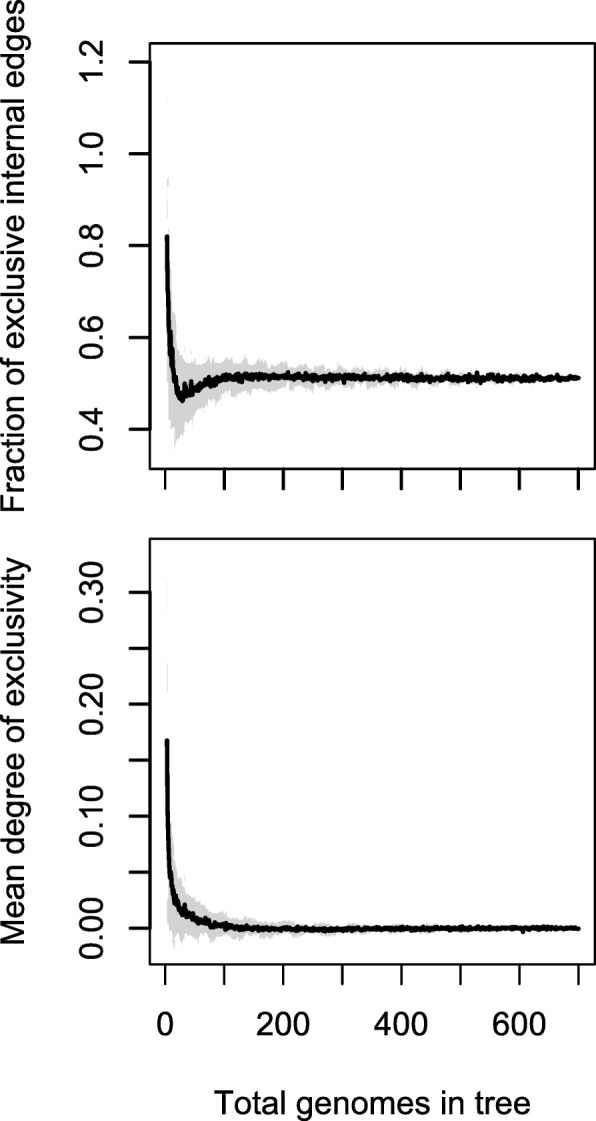
Exclusivity is unlikely to be the result of subsampling Streptomycetaceae genomes. Black lines show average measures of exclusivity for all clades on the pan-genome UPGMA tree for Streptomycetaceae, with ± one standard deviation indicated by the surrounding gray region. Clades are considered exclusive if their exclusivity score determined from the core-genome is greater than zero. Except for very small subsamples, exclusivity quickly plateaus with increasing sample size, suggesting that the existence of exclusivity is not the result of subsampling the total population of genomes

Exclusivity is potentially contingent upon the set of organisms included in a study, since groups can lose their exclusivity if a new strain is discovered that is closely related to a subset of organisms in two non-nested exclusive groups. Therefore, to assess whether the large number of exclusive taxa we identified could be an artifact of under-sampling the total population of Streptomycetaceae, we analyzed increasingly larger random subsets of the 701 genomes, using the original matrix of average patristic distances to recalculate exclusivity scores for clades in a re-computed pan-genome tree for each subsample of genomes. Whether considering the average exclusivity score or the proportion of clades that are exclusive, exclusivity quickly converges as more genomes are sampled (Fig. 4), plateauing at around 100 genomes. These findings suggest that further increasing the sampling of genomes would continue to add additional exclusive clades at the same rate that exclusive clades were disrupted, implying that the observed degree of hierarchical structure is not simply an artifact of under-sampling.

### Absence of a clear transition at which to define the species rank

**Fig. 5:**
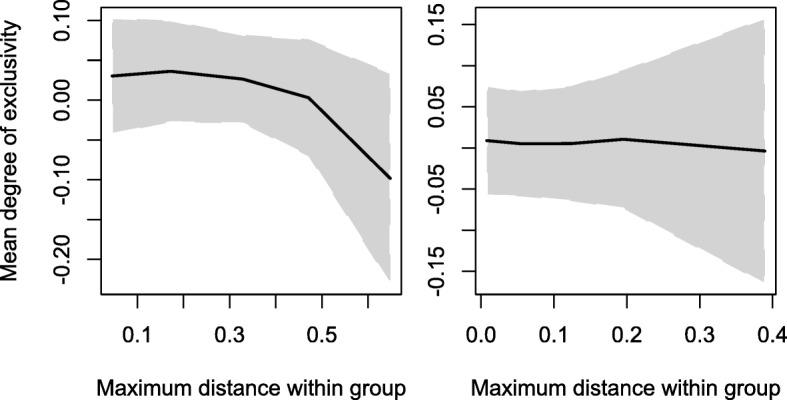
Absence of an abrupt transition for denoting the species rank. The average degree of exclusivity based on the core-genome average patristic distances (y-axis) is shown for clades with increasing breadth (x-axis) on the pan-genome tree. The lack of a relationship between exclusivity and clade diversity (maximum in-group distance) in Streptomycetaceae (left) or *Bacillus* (right) shows that there is no phase transition in exclusivity that could serve as an objective species-level ranking criterion. The gray region shows ± one standard deviation from the average exclusivity at each maximum in-group distance

Having identified exclusive groups, we wished to explore whether there is an objective basis for treating some exclusive taxa as species. The fact that existing species names often match clades with relatedness above a certain threshold has previously been used to argue for an objective species boundary [13, 52]. However, this reasoning is suspect because defining a cutoff for the species rank based on what has previously been considered a species amounts to circular reasoning. To explore the objectivity of the species rank, we tested the hypothesis that there might be a sharp transition in the degree of exclusivity as a function of clade depth (i.e., increasing maximum in-group distance). Baum and Shaw [67] hypothesized that exclusivity would not apply within species but would emerge as one considered progressively more inclusive clades at or above the species rank. As can be seen in Fig. 5, this hypothesis is incorrect. Instead of an increase in exclusivity these data show a gradual decline in the degree of exclusivity with increasing in-group distance. Furthermore, there is no sudden step in the exclusivity function and, thus, no reason to believe that the species rank can be associated with an abrupt change in patterns of relationship. Therefore, while exclusive taxa are real, there does not appear to be any objective criterion based on genealogical concordance for denoting the species rank within bacterial genera.

### Exclusive taxa can be ranked as species using sequence similarity cutoffs

**Fig. 6:**
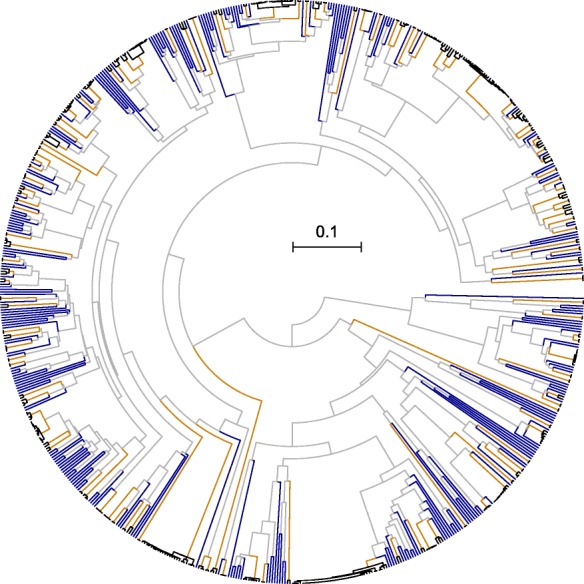
Core-genome tree depicting species groups defined in Streptomycetaceae with our methodology. A UPGMA tree was constructed using the average of 157 matrices containing the pairwise patristic distances derived from each core-gene tree. Exclusive groups were defined according to the same matrix of average patristic distances for the 701 genomes. We then delineated as species the largest exclusive groups whose members met the joint ANI/AF criterion with a single-linkage approach. Singleton species are represented by blue leaves, whereas species with multiple genomes appear as a group of black leaves subtended by a brown edge

In bacterial systematics, it has been common to associate species with a threshold of sequence similarity, often based on a single reference gene region (i.e., rDNA or *rpoB*). More recently, it has been argued to use a combination of both a genome-wide ANI of ≥96.5% and an AF of ≥60% [52, 68]. Here, we applied this combined ANI/AF criterion, equating species with the largest exclusive groups whose members satisfy the ANI/AF threshold with respect to one another (treating singleton tips as trivially exclusive). As above, we used a matrix of average patristic distances to calculate exclusivity and a UPGMA tree constructed from this matrix to define clades (Fig. 6). The ANI/AF threshold was applied in two ways: complete-linkage (members of a species meet the criteria with respect to all other members) and single-linkage (each member of a species meets the criteria with respect to at least one other member). Among the 500 exclusive groups detected, the complete- and single-linkage criteria assigned 374 and 372 to the species rank, respectively, of which 250 and 249 are singletons. The largest number of genomes assigned to a single species was 38. The large proportion of singleton species reflects the stringency of the joint ANI/AF criterion and the fact that the genus *Streptomyces* is evolutionarily ancient [35]. Notably, the addition of an exclusivity criterion does not result in over-splitting of species: among all pairs of genomes that meet the ANI/AF threshold, only 0.8% and 0.4% (for complete-linkage and single-linkage, respectively) were split into different species by considering exclusivity.

### Similar results are observed in *Bacillus*

To determine whether our conclusions were applicable beyond Streptomycetaceae, we conducted the same set of analyses on a set of 1,586 genomes belonging to *Bacillus*. This resulted in a matrix of 140,300 COGs shared by at least two strains and 155 core-genes shared by all sampled *Bacillus* genomes. In comparison to *Streptomyces*, *Bacillus* was more redundantly sampled, with four named species being represented by more than 100 genomes each. Nonetheless, analyses of these *Bacillus* genomes yielded similar results to Streptomycetaceae (Fig. 5, Additional file 1: Figures S2-S3). Again, we observed substantial congruence between trees constructed from the core-genome and pan-genome data partitions (Additional file 1: Figure S4) and the probability of two random trees being this similar by chance is vanishingly small (10^− 1712^). On the pan-genome tree, 447 clades (28.2%) were exclusive according to the matrix of average core-genome patristic distances. Similarly, a UPGMA tree based on average patristic distances had 922 (58.2%) exclusive clades. These lower percentages (than Streptomycetaceae) mainly reflect a lack of exclusive groups in densely sampled clades of closely related genomes. Only 4 tips were not part of any exclusive group and, as was the case for Streptomycetaceae, all of these tips could be considered singleton species due to their substantial distance from any other genome in the set.

As with the Streptomycetaceae, we applied automatic delimitation of species in *Bacillus* based on exclusivity using the core-genome patristic distances combined with the same joint ANI/AF threshold. This approach identified 330 species with complete-linkage and 219 with single-linkage, of which 168 and 107 were singletons (Additional file 1: Figure S5). The average number of genomes per species for the single-linkage case in *Bacillus* (7.2) is higher than Streptomycetaceae (1.9) reflecting the existence of a higher average pairwise similarity among *Bacillus* strains, especially in the most densely sampled clade (Additional file 1: Figure S5, right side). Furthermore, the *Bacillus* data set includes many more pairs of potential conspecifics (i.e., strains whose similarity is greater than the ANI/AF threshold) that were split into separate species by the exclusivity criterion: 58.5% and 20.5% by complete-linkage and single-linkage, respectively. This discrepancy reflects the relatively dense sampling of closely related *Bacillus* genomes, which results in a large number of clades with exclusivity scores very close to zero. Indeed, if we permit species groups with exclusivity scores slightly less than zero (> − 0.01), we find that only 0.8% of potential conspecifics are assigned to different species by single-linkage. This looser definition of exclusivity prevents minor discrepancies in the distance matrix from breaking exclusivity, and resembles relaxed definitions of clades and the core-genome that have been applied in other studies [62, 69]. Notwithstanding these differences between Streptomycetaceae and *Bacillus*, our analyses suggest that exclusive taxa are widespread among bacteria and illustrate that our approach for automatically delimiting species groups is generalizable.

## Discussion

We have demonstrated that numerous exclusive bacterial taxa exist when the entire genome is considered, despite the fact that few clades are shared by all gene trees (i.e., concordance factors are rarely 1.0). This observation is in line with the claim that there is considerable horizontal gene flow in bacteria but that this gene flow is insufficient to overpower the strong signal of vertical inheritance. If the existence of taxa is contingent on strictly vertical inheritance of all genes then bacterial taxa certainly do not exist. However, we have shown that if bacterial genomes are considered holistically as composites of many genes then bacterial taxa are widespread. We believe that defining taxa by the average history of many genes detects taxa in cases where there is an overall signal of vertical inheritance and hierarchical structuring of traits, which are qualities that make taxa useful constructs. Such conditions hold for many groups within *Streptomyces* and *Bacillus*, justifying their recognition as taxa using our genome-wide perspective.

Species of bacteria have been traditionally defined based on having shared genotypic and phenotypic characteristics that distinguish them from other groups. The International Committee on Systematics of Prokaryotes oversees a set of standards for establishing a new species that require a representative isolate of the bacterium [70]. These standards have been criticized as impractical since they are laborious to apply and many bacteria have yet to be cultured in the laboratory [68]. Thus, microbiologists have begun to adopt genomic species delimitation procedures, such as ANI, that can be applied to thousands of genomes from uncultured organisms. While ANI is useful for assigning a new genome to the same species as an existing labeled genome, a new genome may map to two different labels and there is no agreed upon method (e.g., single-linkage clustering) for assigning species groups given a large number of existing genomes. More recently, a tree-based approach based on relative evolutionary divergence has been suggested [71], which corrects for varying rates of evolution and allows for flexible cutoffs to preserve existing taxonomic names. Here we recommend a similar phylogenetic approach, with the additional constraint that taxonomic groups have the property of exclusivity. This ensures that species are worthy of inclusion in the taxonomic system and avoids recognizing as species groups of organisms that have conflicting relationships for substantial parts of their genomes [27].

Although our analyses support the existence of many exclusive taxa, they contradict the claim that the species *rank* has objective reality. The existence of an objective species rank would require that some taxa have a defining characteristic that separates them from more inclusive (e.g., genera) or less inclusive (e.g., subspecies) taxa. Although it has sometimes been postulated that such an objective ranking criterion exists [13, 72], we found a continuous gradation in exclusivity, which rules out any simple ranking criterion based upon genealogical relatedness. Combined with the consistent failure of systematics to identify a non-genealogical criterion that would apply only to one clade in a system of nested clades, and would make those clades comparable across diverse branches of the tree of life, we conclude that there is no objective way to consistently discriminate among the exclusive groups that we treat as genera, species, or subspecies. That is, species *groups* may be real, but the species *rank* is not. This follows from the fact that there is a continuous decrease in the rate of horizontal gene transfer as bacteria diverge, which would not be expected to generate sharp discontinuities in gene flow [21].

If the species rank lacks biological reality, should we get rid of species entirely, as advocated by Mishler [73]? We would like to suggest that, given the many practical uses of taxonomy, we should continue to designate one level in the taxonomic hierarchy to serve as the primary name-carrier, the species in the Linnaean system. In the absence of any objective ranking criterion it is still reasonable to assign some exclusive groups to the rank of species, provided it is understood that the “species” is not an objectively real unit [29]. That is, species cannot be treated as a base unit of evolution, and species counts cannot be used as absolute or relative measures of diversity. In many ways, applying a conventionally-defined species rank is easier for bacteria than macro organisms given the long history of using similarity-based conventions to delimit bacterial species. Thus, it becomes possible to consistently define bacterial species as the largest (i.e., most inclusive) exclusive groups of organisms whose members lie within a chosen level of sequence similarity.

As we illustrated here, delimiting species is methodologically straightforward after deciding on a similarity threshold. We recommend inferring a summary tree from a matrix of average pairwise patristic distances derived from core-genes, which should approximate genome-wide relatedness. Then exclusive groups on this tree whose members satisfy the joint ANI/AF criterion, or any other threshold of choice, can be considered species. It is debatable whether a single-linkage or complete-linkage approach is preferable for connecting members of a species, but we lean towards single-linkage due to the ease of including newly sequenced genomes without splitting existing species groups. While this method results in the recognition of 372 distinct species among the 701 Streptomycetaceae genomes, and 219 species among 1,586 *Bacillus* genomes, such high numbers align with the great geological age of many bacterial genera [35] and with prior arguments that taxonomic approaches originally created for multicellular eukaryotes have massively underestimated microbial diversity [74]. Notably, it required around 100 genomes before the proportion of groups showing exclusivity stabilized. This indicates that modest sampling efforts can successfully identify many exclusive taxa, although there is a chance that these groups will lose their property of exclusivity once more genomes are sampled.

Although we advocate that an effort be made to only recognize exclusive groups as taxa of any rank, including species, we are less committed to the strict use of any particular similarity-based ranking criterion. Since ranking cutoffs are artificial anyway, it does not seem important that they be applied too rigidly. In particular, the decision as to which exclusive clades to rank as species should take into account practical factors such as clinical relevance, ecological data, historical precedent, and traditional taxonomic practice [52]. We believe it is important to have general standards for delimiting species based on having a largely shared evolutionary history and being substantially diverged from other species, noting that one can always use subspecific ranks to delineate sets of strains with group-specific characteristics that are important to distinguish for medical or applied purposes. For some bacterial lineages it may prove feasible to rank exclusive taxa as species based on evidence of high internal and low external rates of HGT [7], though we are skeptical that this approach will consistently assign only one nested clade to the species rank or that the groups ranked by this criterion will be comparable across taxa with very different natural histories. Thus, in most cases, the automated species delimitation procedure that we employed here will provide a reasonable first-pass classification that could be modified later according to additional practical or ecological considerations.

## Conclusions

In this study, we showed that exclusive taxa exist in *Streptomyces* and *Bacillus* when the genome is considered as a whole despite genealogical discordance. Our measure of exclusivity is based on genome-wide relatedness, which we here estimated based on the pairwise patristic distance on each gene tree averaged across gene trees. This strategy allowed us to show that horizontal inheritance (i.e., HGT) has not overwhelmed the signal of vertical inheritance in these two bacterial groups and provides a good initial basis for delimiting species. In the future, however, the exclusivity criterion could be applied using alternative measures of the degree of relatedness. For example, instead of just averaging patristic distances across genes, one might first rescale branch lengths to account for rate heterogeneity across genes and perhaps enforced ultrametricity to ensure a closer connection between pairwise distance and time since common ancestry, the most direct measure of the degree of relatedness. Furthermore, it might be possible under certain assumptions to estimate exclusivity from other distance metrics, including the proportion of shared alleles, ANI, or gene content similarity.

As more genomes are made available it will become feasible to determine whether our findings apply to other well-sampled prokaryotic genera. Furthermore, we hope that a similarly unbiased sampling of multicellular eukaryotic genomes will become available. This would be helpful to test the widely held, but as yet untested, presumption that sexual eukaryotes form clearer natural species than bacteria [72]. We would speculate that, even though HGT may be less common in eukaryotes, it, and other kinds of reticulate evolution do occur [20, 21, 75], meaning that a genome-averaging approach will still be needed to delineate taxa across the tree of life. Thus, we predict that, despite HGT playing a greater role in bacteria and sexual hybridization being more prevalent in eukaryotes, the core patterns will be the same in all groups: exclusive taxa exist when the genome is considered in its entirety, but the choice of which to rank as species can only be made by falling back on practical factors and/or thresholds established by convention.

## Additional file

Additional file 1: Supplemental Figures S1-S5. (PDF 143 kb)
