# Supplementary material for: Exclusivity offers a sound yet practical species criterion for bacteria despite abundant gene flow
Source: BMC Genomics. 2018 Oct 3;19:724. doi: 10.1186/s12864-018-5099-6 (PMC6171291; doi:10.1186/s12864-018-5099-6)
Supplement: Supplementary file 1 — Supplemental Figures S1-S5. (PDF 143 kb) [file 12864_2018_5099_MOESM1_ESM.pdf]

Supplementary Material for "Exclusivity offers a sound yet practical species criterion for bacteria despite abundant gene flow" by Erik S. Wright and David A. Baum

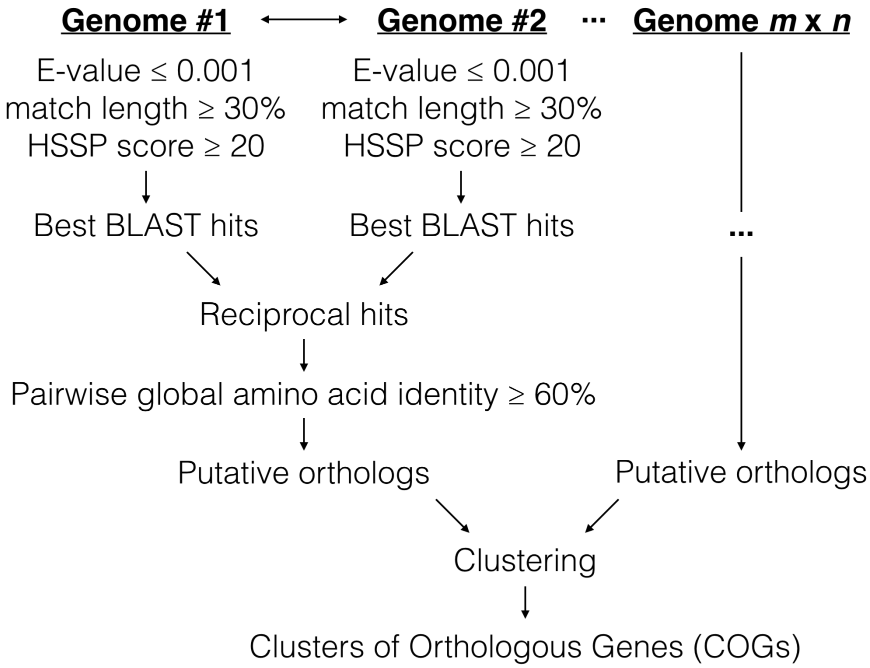

Figure S1. Workflow for identifying clusters of orthologous genes (COGs). Pairwise protein BLAST was conducted for *n* genomes against every other genome as both the query and subject. The top reciprocal hits were aligned and filtered at 60% pairwise amino acid identity. The filtered homologs were clustered into COGs, which were then used to define the genes composing the core-genome and pan-genome (see Methods).

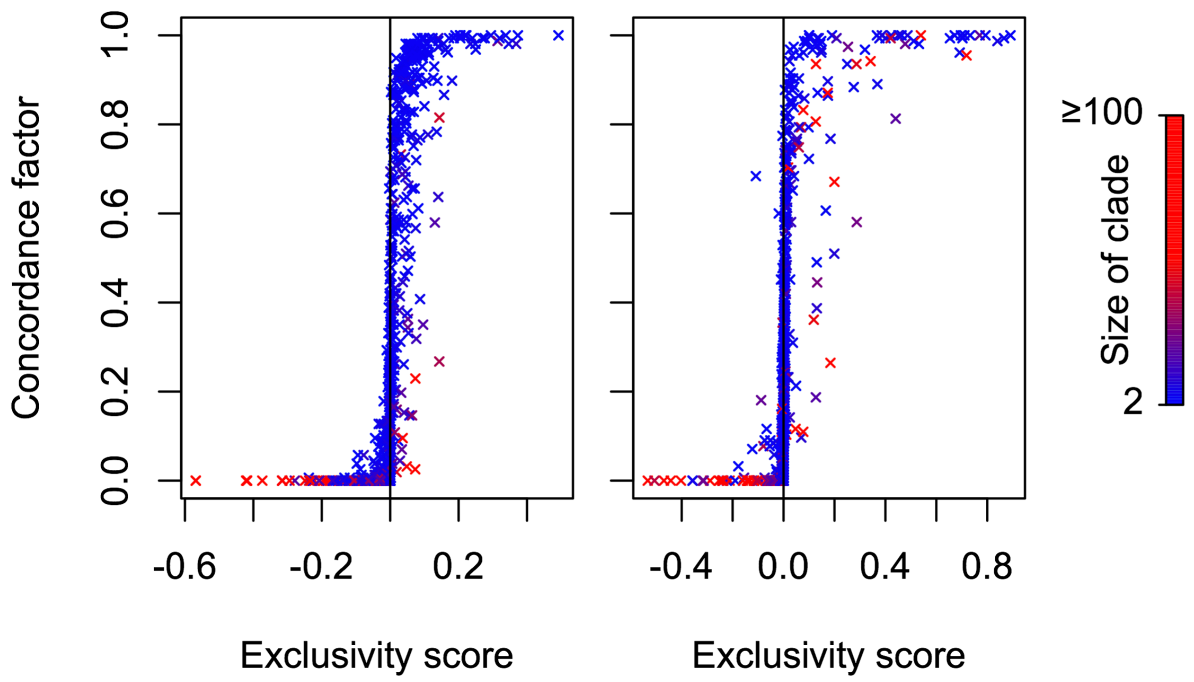

Figure S2. Relationship between concordance factor and exclusivity in Streptomycetaceae (left) and *Bacillus* (right). As a rule, clades with positive exclusivity scores tended to have higher concordance factors. Exceptions to this rule were generally large clades for which exclusivity could be positive despite a low concordance factor.

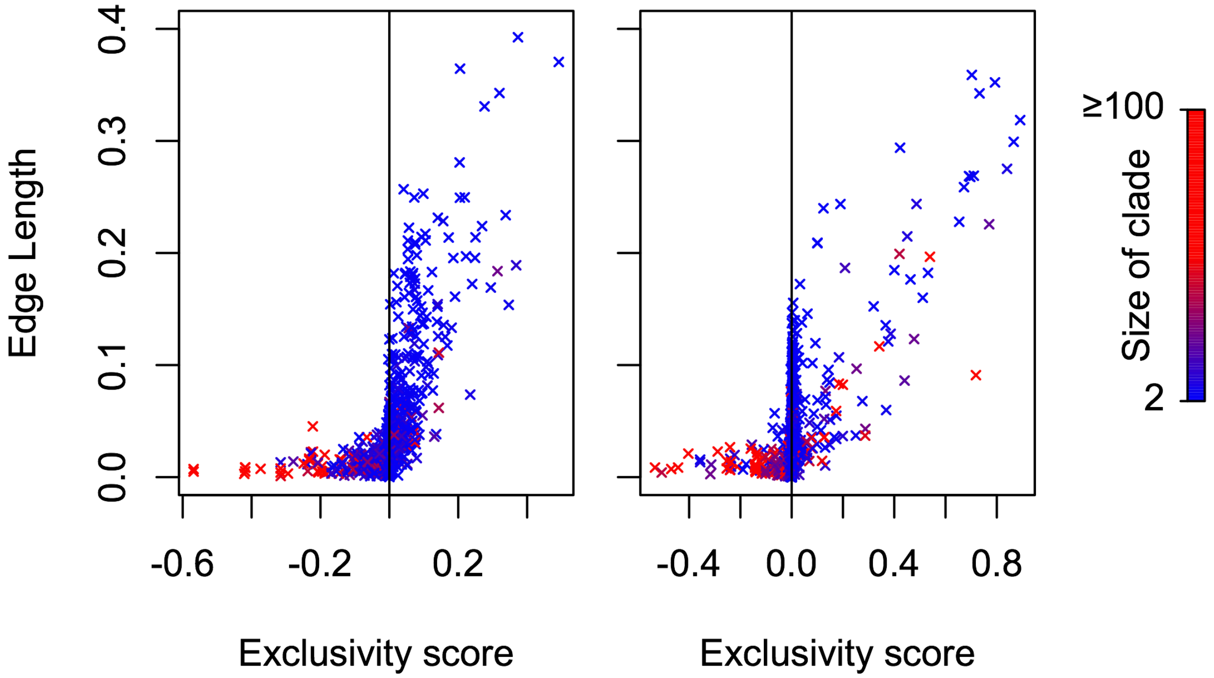

Figure S3. Edge length increases with increasing exclusivity in Streptomycetaceae (left) and *Bacillus* (right). As anticipated, edge lengths tended to increase along with the exclusivity (based on core-genome patristic distances) for internal edges of the tree constructed from gene content similarities. Hence, exclusivity increases as a clade becomes separated by a longer edge on the tree. This is consistent with the notion that taxonomic groups can be identified as clusters of strains separated from all other strains by a relatively long branch.

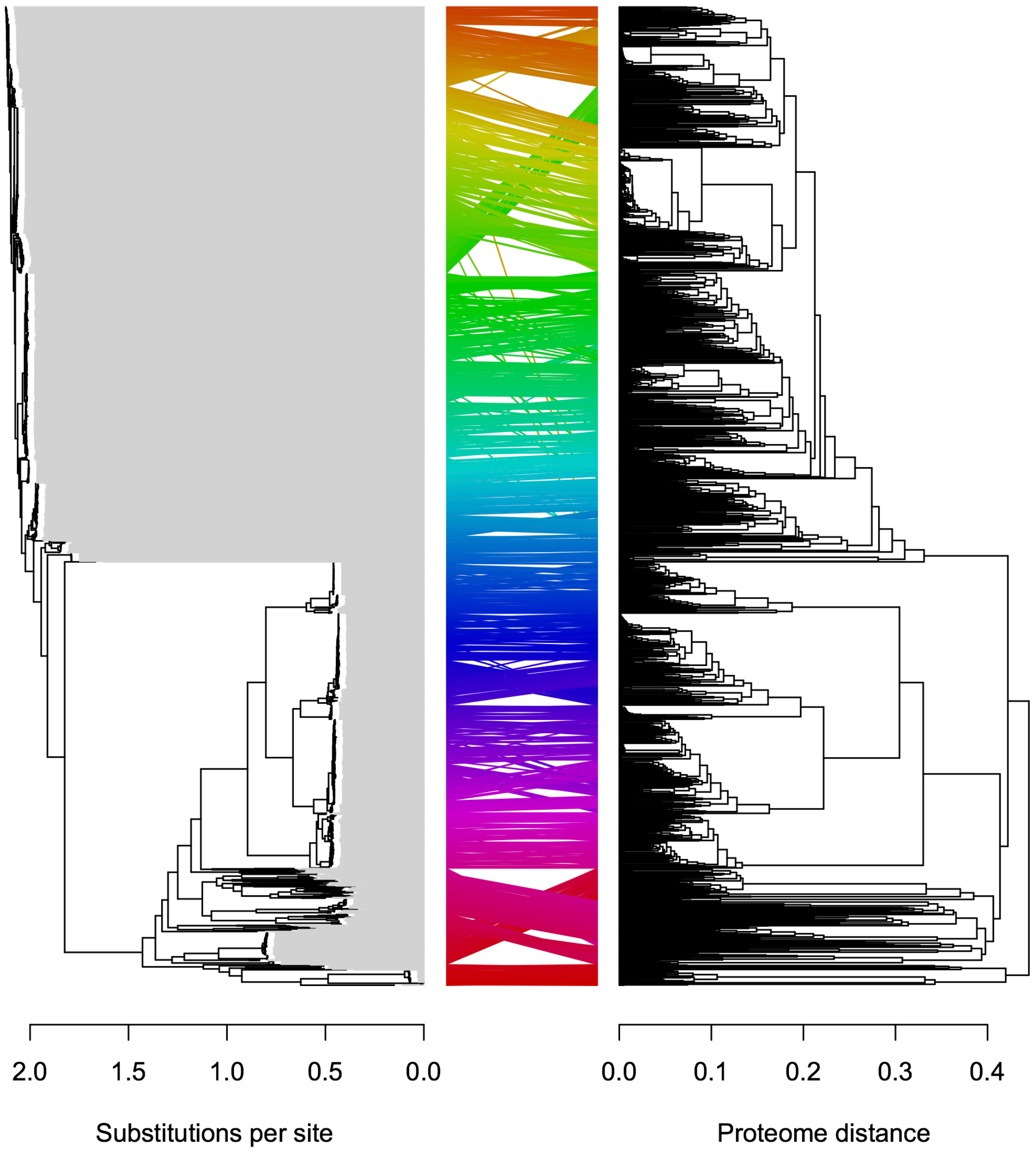

Figure S4. Concordance between the core-genome and pan-genome trees representing relationships among 1,586 *Bacillus* genomes. In agreement with Streptomycetaceae (Fig. 2), the core-genome tree (left) and pan-genome tree (right) both share the same majority phylogenetic signal.

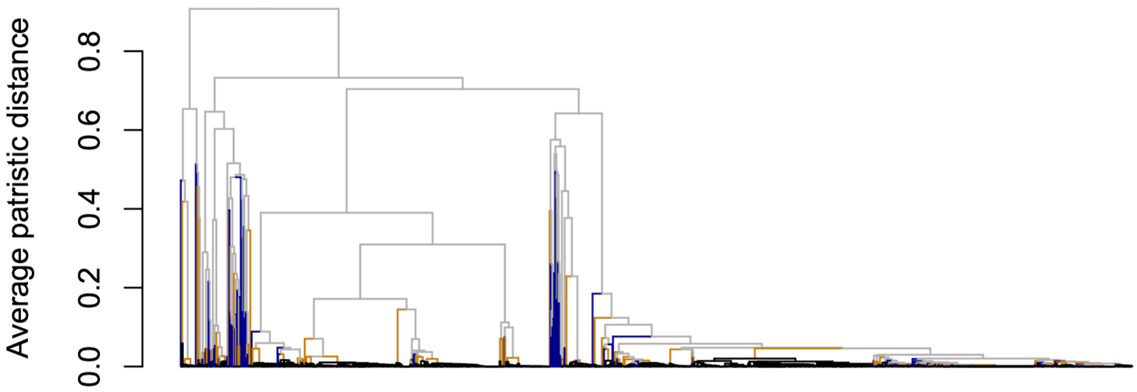

Figure S5. UPGMA tree depicting species groups defined in *Bacillus* with our methodology. A UPGMA tree was constructed using the average of 155 matrices containing the pairwise patristic distances derived from each core-gene tree. Analogous to Fig. 6, exclusive groups were defined according to the same matrix of average patristic distances among the 1,586 genomes. We then delineated the largest exclusive groups as species whose members met the joint ANI/AF criterion with a single-linkage approach. The resulting species are denoted by brown edges separating black leaves, or blue leaves in the case of singleton species.
